# A Comprehensive Review of the Historical Description of Spine Surgery and Its Evolution

**DOI:** 10.7759/cureus.54461

**Published:** 2024-02-19

**Authors:** Tania Mamdouhi, Victoria Wang, Alexandra C Echevarria, Austen Katz, Matthew Morris, Gabriel Zavurov, Rohit Verma

**Affiliations:** 1 Orthopedic Surgery, Zucker School of Medicine at Hofstra/Northwell, Hempstead, USA; 2 Orthopedic Surgery, University of Michigan, Ann Arbor, USA; 3 Orthopedic Surgery, University of Connecticut, Storrs, USA; 4 Orthopedic Surgery, North Shore University Hospital, Manhasset, USA; 5 Orthopedic Surgery, Spine Surgery, North Shore University Hospital, Manhasset, USA; 6 Orthopedic Surgery, Northwell Health, Manhassett, USA

**Keywords:** minimally invasive spine surgery, spinal fusion, laminectomy, history of spine surgery, spine surgery

## Abstract

Major strides in the advancement of spine surgery came about in the 21^st^ century. However, the extensive history of spine surgery can be traced back to long before this time. A clear description of the journey from a primitive yet accurate understanding of the human musculoskeletal system to today’s modern aspects of spinal techniques is lacking. A narrative literature review was conducted to elucidate where spine surgery began and the techniques used that evolved over time. This review was conducted using PubMed and Google Scholar. Search terms used included “history of spine surgery," “evolution of spine surgery," “origins of spine surgery," “history of laminectomy," “history of spinal fusion," “history of lumbar interbody fusion," “minimally invasive spine surgery," and “navigation in spine surgery." We highlight the evolution of the basic understanding of anatomy and non-surgical and surgical techniques, including bracing, laminectomy, discectomy, and spinal fusion. The current evolution and integration of minimally invasive techniques, lumbar interbody fusion techniques, robotics, navigation, and motion preservation are discussed, as these are the major areas of focus for technological advancement. This review presents an overarching synopsis of the events that chronicle the progress made in spine surgery since its conception. The review uniquely contributes to the growing body of literature on the expansion of spine surgery and highlights major events in its history.

## Introduction and background

To understand the origins of modern-day spine surgical and non-surgical techniques, a look must first be taken at the contributions of ancient society that aided in understanding the anatomy and pathology of the spine. A narrative literature review was conducted using PubMed and Google Scholar. Search terms used included “history of spine surgery," “evolution of spine surgery," “origins of spine surgery," “history of laminectomy," “history of spinal fusion," “history of lumbar interbody fusion,” “minimally invasive spine surgery,” and “navigation in spine surgery." Articles and publications were reviewed by the authors for their relevance and accuracy. For newer technologies and surgical techniques, an emphasis was placed on predominantly including references published within the last 10 years to accurately highlight current developments.

Ancient spinal intervention has previously been categorized into four major time periods: Egyptian and Babylonian, Greek and early Byzantine, Arabic, and Medieval [1]. The Egyptian and Babylonian periods offer the earliest written mention of surgery in the Edwin Smith papyrus, written after 1700 BC, where 48 cases of spine and cranium injuries are described [1]. The Greek and early Byzantine periods are best known for their substantial contributions to surgical innovation, with Hippocrates, who is often referred to as the “Father of Spine Surgery," and Galen of Pergamum being essential contributors [2]. The Arabic and Byzantine era, approximately 750 AD to 1200 AD, was a predominantly dormant period in surgical exploration, mainly characterized by codifying and translating works from the Greek and Roman periods [1]. Introductions to antiseptic and hemostatic techniques flourished in the medieval period, with Guy de Chauliac having a predominant influence in the 14th century regarding his work on anesthetics and anatomical explanations [1]. 

History then takes a massive leap into the late 1930s, when Jules Guerin was the first to use surgical intervention in treating scoliosis [3]. He integrated percutaneous myotomies of vertebral muscles with an external bracing technique, combining the treatment modalities available at the time [3]. Over time, surgical approaches to the spine have expanded with the advancement of surgical tools, and success with newer approaches has become possible. Today, the use of minimally invasive techniques, navigation, and robotics is the new frontier for accelerating spine surgery into the future. Throughout this review, we highlight the major historical contributions within each sector of spine surgery from a global perspective.

## Review

Historical background

Within his overall contributions to the field of medicine, Hippocrates took particular interest in the field of orthopedics, where his influence on clinical practice is still apparent today [2]. His treatises on Joints and on Nature of Bones are the first recorded in history to provide details on spinal anatomy, disease, and suggestions for treatment techniques [4]. Most notably, these works describe his study and experimentation with deformities of the spine, which we now understand to be interpretations of scoliosis [3]. The Hippocratic ladder and bench, which have been the basis for modern-day traction strategies for non-surgical bone fracture and scoliosis correction, are described. For example, the Vertebral Axial Decompression (VAX-D) (approved by the Food and Drug Administration (FDA) in 2007) is designed to apply distraction tension and non-surgically decompress the spine and intervertebral discs [5]. Both the bench and the VAX-D apply tension to the spine; however, the VAX-D incorporates a computer program for real-time feedback, minimizing the concern for reflex paravertebral muscle contraction [5]. 

Galen of Pergamum, a student in the Hippocratic school of thought, had great influence in the 2nd century A.D. as he was among the first to modify the Hippocratic technique [6]. While Hippocrates is known to have set the basis for traction techniques, Galen set the basis for bracing. He discussed 24 vertebrae (specifically 7 cervical, 12 thoracic, and 5 lumbar), generated the terminology for scoliosis, lordosis, and kyphosis, and was the first to design and experiment with chest binders and jackets to treat scoliosis [3,7]. Galen’s understanding of pathology allowed him to tailor the application of traction and compression to each patient’s specific deformity, which was lacking in the work Hippocrates carried out [8].

Evolution of bracing techniques

**Figure 1:**
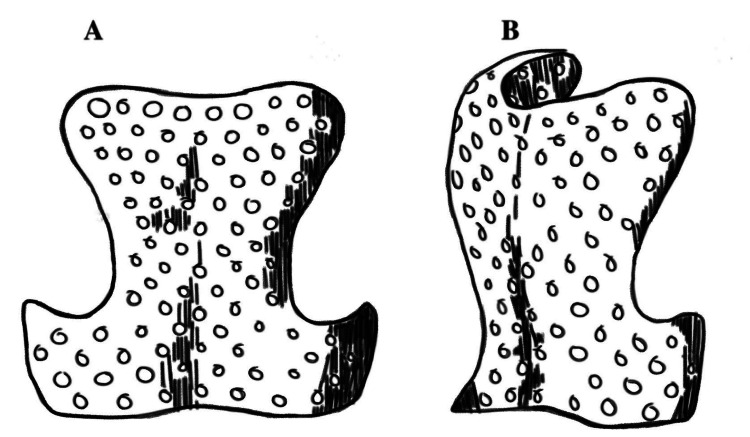
Illustration of the anterior and posterior plates of the padded iron corset Ambroise Paré would use for the treatment of scoliosis. Reproduced from: Williams AN, Williams J. 'Proper to the duty of a chirurgeon': Ambroise Paré and sixteenth century paediatric surgery. J R Soc Med. 2004;97(9):446-449. doi:10.1258/jrsm.97.9.446. Image published under Creative Commons License BY 4.0.

The use of bracing for spinal deformity correction began in the 1500s with Ambroise Paré, a French army surgeon. Paré would apply direct pressure and traction to the spine, which would be followed by the application of a padded iron corset that he had designed for the correction of spinal deformity (Figure 1). He was the first to emphasize the importance of bracing before complete skeletal maturity after noting unsuccessful outcomes in those with mature spinal anatomy [6].

Nicholas Andry De Bois published L’Orthopedie in 1741, describing treatment for scoliosis using a method of bracing [3]. The publication not only established the title Orthopedics but also describes prevention methods for spinal deformity in pediatric patients [3]. The long-term use of a padded brace to correct scoliosis was recommended by Kohler [9]. Tarpada et al. later designed the Jurymast Brace, which had the notable advantage of allowing patients to move upright [3]. Increased mobility made long-term bracing much more manageable, which was revolutionary compared to braces from the prior two centuries.

At the onset of the late 1800s, the Sayre Jacket was created by Lewis Sayre. Contrary to De Bois, Sayre believed musculoskeletal imbalance was the root cause of spinal disorders [10]. He recommended targeted muscular strengthening to correct such imbalances, which can be thought of as a modern-day form of physical therapy [10]. His infamous Sayre Jacket was made of plaster of Paris body casts, which he would apply after suspending patients from their arms and heads. The casting would extend to just below the anterior superior iliac spine, completely encasing the patient’s spine [11]. Although it is debated whether his bracing technique was truly efficacious, his method of bracing can be considered the start of modern bracing methods for spinal deformity [10].

Laminectomy and discectomy

Major advancements prior to the 19th century were centered around non-surgical approaches; it was the development of posterior and later anterior surgical approaches to the spine that opened the door to surgical intervention [12]. The first thoracic laminectomy was performed by Henry Cline in London in 1814; however, the patient died three days after surgery, which was attributed to the severity of the traumatic injury [12]. A laminectomy is the removal of all or part of the lamina, which is the vertebral bone that covers the spinal cord and nerve roots posteriorly, to decrease pressure. The first successful laminectomy was not performed until 1828 by Alban Smith [13]. Elsberg completed the first cervical laminectomy in 1926 for the removal of an intramedullary spinal tumor [14]. Laminectomy was the major method for decompressing degenerative conditions of the cervical spine until techniques for laminoplasty and, eventually, posterior spinal fusion were created. A laminoplasty is a procedure performed to relieve pressure on the spinal cord by removing part of one side of the lamina at the affected level. High rates of complications, such as progressive cervical kyphosis and spinal cord injury, made laminectomy dangerous, thus necessitating a new methodology. The application of spinal fusion to correct kyphosis has been successful. However, the advent of laminoplasty offered a surgical alternative at the time [15]. Laminoplasty was first described in Japan for degenerative spinal canal stenosis and ossification of the posterior longitudinal ligament. The most notable technique is the open-door laminoplasty introduced by Hirabayashi et al., in 1977, which addressed issues with post-laminectomy instability and malalignment [15,16]. Open-door laminoplasty involves the formation of bony gutters along the medial margins of the facets, which on the unaffected side operate as a hinge joint. Specific laminoplasty plates with screws are routinely used to secure the hinge in place [17]. The displaced posterior bony process is then stabilized via sutures to the musculature and capsule surrounding the facets to prevent closure of the laminar door [17].

Integration of discectomy into laminectomy procedures became progressively more popular as an understanding of intervertebral disc disease developed [18,19]. A discectomy is used to remove part of a damaged disc that has herniated and is irritating and compressing the associated nerves. The first discectomy was done by Fedor Krause in 1908 in Berlin and involved a laminectomy for access [18,19]. The tissue removed was mistaken for an osteochondroma at the time, a common misconception. It was not until 1934, when Mixter and Barr performed a discectomy in the lumbar spine, followed by pathologic confirmation of disc tissue removal, that the true utility of discectomy was considered [20]. Mixter and Barr presented their observed correlation between disc prolapse and clinical signs of nerve root and cord compression and advocated the use of surgical discectomy for relief [19].

Spinal fixation and fusion: surgical history

It quickly became clear during the advancement of spine surgery that methods of fixation, especially internal methods, should not replace the need for spinal fusion, as it was the most crucial component for spinal deformity correction [21]. The first successful internal fixation technique was seen in 1891 when Hadra used a silver wire loop wrapped around the spinous processes of C6-7 in a figure eight orientation to successfully treat cervical fractures [21]. Hadra’s use of wiring in the cervical spine was followed by a method of interspinous wiring first described by Rogers in 1942, and Callahan et al. also described facet wiring in 1977 [22,23].

Lange was regarded as the first to offer a fixation technique for patients with scoliosis in 1909 [3]. His method of fixation involved celluloid bars, steel, and silk wiring, with the incorporation of Sayre’s plaster jacket for post-operative short-term stabilization [3]. His method was shown to halt the progression of disease; however, it was later abandoned due to the corrosion of the steel implants.

In 1909, Hibbs published his technique of knee arthrodesis, or joint fusion, which he then modified for posterolateral spinal arthrodesis in 1911 [24]. His technique was the first report of spinal fusion and involved transposing the spinous processes caudally via a partial fracture to bridge the gap between vertebrae [24]. Mixter and Osgood described a fixation technique for atlantoaxial instability around the same time as Hibbs’s publications. Via a posterior approach, they used silk sutures anchored on the spinous process of the axis to fixate the instability after manual reduction [21]. Later, in 1931, Hibbs et al. combined the use of posterior spinal arthrodesis with an established method of cast immobilization called the "turnbuckle," obtaining a 70% rate of maintenance or improvement compared to the pre-operative state [25]. Originally created by Lovett and Brewster, the turnbuckle was adopted for post-operative immobilization. Risser later modified the cast to make it lighter and improve the maintenance of trunk alignment [6]. In 1939, Gallie upgraded the method of spinal fusion to one that was widely used throughout the rest of the 20th century using autografts that were held in place by sublaminar wiring between the C2 spinous process and the C1 arch [16,26].

Access to neck structures through an anterior surgical approach arose at the end of the 1890s, initially for non-spinous pathologies [16]. The advent of an anterior approach by Abbott in 1952 minimized the risk of cervical spine surgery by circumventing the spinal cord and vertebral arteries [27]. Anterior cervical discectomy and fusion (ACDF) was introduced in 1958, separately by Smith and Robinson [28] and Cloward [29]. Both describe creating a plane of cleavage between the carotid sheath and more medial structures to reach the anterior surface of the cervical spine. Many modifications have been made to incorporate newer tools and technologies into this procedure, as it is one of the most fundamental procedures conducted by spine surgeons today.

Advancements in the use of screws and rods

Methods of fixation using both vertebral and pedicle screws became increasingly popular as technology developed. Screws decreased the need for long-term immobilization without sacrificing stability. The first description of screw placement for lumbosacral fusion was published by King in 1948 [30]. Screws were drilled through both facets, with placement parallel to the inferior border of the lamina and perpendicular to the facet joints [31].

Nine years later, Boucher published his method of internal fixation using longer, translaminar pedicle screws. He uniquely described the placement of pedicle screws to improve fixation, having success at multiple levels [32]. In 1970, Orozco and Llovet were the first to use what is now called unrestricted backout plates for anterior cervical spine surgery [33]. Expanding upon the pedicle screw technique, Raymond Roy-Camille, in 1970, used posterior plating with screws positioned sagittally through pedicles and articular processes [31]. Roy-Camille’s pedicle screw fixation technique laid the foundation for the development of transpedicular devices.

Prior to Roy-Camille’s contributions, Harrington created the design for the Harrington Rod, the first method of internal fixation and realignment [34,35]. It was in 1955 that the original Harrington was developed using a customized rachet rod and hook instrumentation for manual open correction [34,35]. Integration of the transpedicular screw into the Harrington Rod design was published in 1969 by Harrington and Tullos [36]. Although revolutionary, the system posed concerns due to the need for post-operative casting as well as the frequent loss of normal lumbar lordosis, leading to what was coined ‘flat back syndrome’ [37]. 

Eduardo Luque adjusted Harrington’s rods in 1977 using a segmental sublaminar wiring technique with flexible rods and wires to anchor a metal pin [34]. This segmental approach allowed for the elimination of external fixation with post-operative casting and provided improved sagittal adjustment [34]. In 1978, Hopf et al. introduced a segmental hook system that integrated multiple fixation points using hook and rod combinations [38]. This system allowed for three-dimensional (3D) spinal deformity correction in the sagittal, coronal, and rotational planes, which was a major advancement from previous systems. By applying cross-links between the rods at both ends of the construct, the two rods were converted to a more stable unitized quadrilateral frame.

Of the contributions to cervical spine stabilization, the largest is considered to be from Roy-Camille with the introduction of the lateral mass screw and plating system via the posterior approach [39]. Lateral mass screws allow for cervical stabilization even in cases where posterior spinal elements are compromised. Magerl et al. modified Roy-Camille’s method in 1987 by applying lateral mass screws to atlantoaxial fixation [40]. Hook plates were fixated using screws placed at a 20-30 degree angle at the junction of the articular process and lamina, avoiding the vertebral artery. An alternative technique using plate and screw fixation of the lateral masses of the atlas and axis was introduced in 1994 by Goel and Laheri with much success [41,42]. Harms and Melcher later introduced a novel technique of atlantoaxial stabilization via polyaxial screws in the C1 lateral masses and C2 pedicle via a posterior approach [43]. The use of pedicle screws for subaxial stabilization eventually became popular due to their superior stability and resistance to screw pullout compared to lateral mass screws at these levels [27].

Several modifications of the original Harrington rod have been introduced more recently, including the Variable Screw Placement (Steffee) system, the Isola system by Marc Asher, the Texas Scottish Rite Hospital (TSRH) system by Richard Ashman and Charles Johnston II, and the Miami Moss system by Shufflebarger and Harms [34,37].

Methods of lumbar interbody fusion

An effective surgical method of neural root and central canal decompression was needed to manage pain, spinal deformity, and progressive neurological disability [44]. Hibbs’ posterolateral fusion approach opened the door for other lumbar fusion approaches, such as interbody fusion, which was a great area of focus throughout the 1900s. Fusion of the anterior spinal elements was originally hypothesized to have a great advantage in increasing fusion success rates, especially of the lumbar spine [44]. With 90% of the articular surface area being on the anterior lumbar spine, targeting this area should improve stabilization. Because bone heals more favorably under compression, fusion success on the anterior surface, where 80% of the spinal load is placed, should be superior [44]. 

An anterior approach to the lumbar spine was first published by Müller in 1906 to treat an abscess secondary to Potts disease [45]. However, poor patient outcomes with this approach and the publication of Hibbs’ approach in 1911 drew focus to posterior spinal access. Capener, in 1932, described a theoretical operative approach to the anterior spine for spondylolisthesis, concluding that the risk was too great to attempt [46]. It was not until 1933 that Burns performed the anterior lumbar interbody fusion (ALIF) for the treatment of spondylolisthesis [47]. With a transperitoneal approach, he inserted a bone graft between the L5 and S1 vertebral bodies [45,48]. In 1934, Ito et al. described an anterior approach to the lumbar spine for the removal of vertebral abscesses [49]. His extraperitoneal method involved an incision in the ventral abdominal wall and retraction of the peritoneal fold containing the abdominal viscera to expose the retroperitoneal space and, thus, the lumbar vertebra. Mercer, in 1936, modified Burns’ method to an approach most like the modern-day ALIF. Through a transperitoneal approach, he would resect the intervertebral disc and place a bone graft in the intervertebral space [45,50]. Many concerns regarding an anterior approach throughout the 1900s focused on muscle damage, ureter injuries, injury to the sympathetic chain, and venous tears [48]. 

Jaslow completed the first posterior lumbar interbody fusion in 1946 [51]. One year after theorizing an anterior approach to the lumbar spine, Capener became the first to perform a lateral approach for thoracolumbar spine decompression [52]. Minimally invasive techniques began opening doors for new surgical procedures, leading to Obenchain performing the first laparoscopic lumbar discectomy in 1991 [53]. Then, in 1999, microendoscopic tubular systems were integrated into lumbar fusion techniques [54]. 

The lateral approach was eventually applied to a minimally invasive technique for lateral lumbar interbody fusion (LLIF) by Pimenta in 2001 [55,56]. LLIF, also known as extreme lumbar interbody fusions (XLIF), begins with the patient being placed in the right lateral decubitus position. A retroperitoneal approach allows access to the intervertebral disc laterally, where discectomy and interbody implant placement are conducted. The procedure is limited to the T12/L1 through the L4/L5 disc spaces. Access to the L5/S1 disc space is obstructed by the iliac crests, limiting the use of XLIF for pathology at these levels [57]. Improved access to the L4/L5 and L5/S1 disc spaces was achieved through modification of the anterior approach to an oblique lumbar interbody fusion technique (OLIF) by Mayer in 1997 [45,58]. With the patient in the lateral decubitus position, the retroperitoneal space is accessed anterior to the psoas. This approach minimizes injury to the psoas muscle itself and, subsequently, to the lumbar plexus, minimizing neurologic injury as well as injury to abdominal structures.

Development of minimally invasive spine surgery

The concept of minimally invasive surgery arose in 1987 with the first laparoscopic cholecystectomy. Minimally invasive spine surgery began with techniques centered around disc disease-relieving resultant neural compression. Chemonucleosis using chemopapain started being used clinically in 1964 by Smith after being discovered in 1941 [59]. Smith’s method involved direct injection into the intervertebral disc, leading to depolymerization of the nucleus pulposus and a resultant reduction in disc height and degree of herniation [59]. Schreiber and Suezawa are known for their contributions to endoscopic spine surgery with the development of a series of cannulas in 1988 [60]. Methods of percutaneous nucleotomy and discectomy followed throughout the late 1900s, eventually progressing to laser-assisted methodologies. These methods were only applicable in specific scenarios, making widespread use difficult with limitations in visualization.

The need for improved visualization sparked the use of microscopes, starting with the microdiscectomy procedures performed by Yasargil and Caspar in 1977 [61,62]. The microscope revolutionized spine surgery, allowing for smaller incisions without compromising visualization. It was later adapted and updated in 1997 to a microendoscopic discectomy (MED) system by Foley and Smith, which was successively modified to generate the microdiscectomy tools used today [61,63]. The system involves using tubular dilators as retractors to increase workspace diameter. A larger workspace allowed for additional procedure tools, opening the door to procedures like laminectomy and foraminotomy [59].

Harms and Jeszenszky were the first to describe a transforaminal lumbar interbody fusion technique (TLIF) in 1998 [64]. His method involved unilateral facetectomy and circumferential fusion [65]. Then, in 2003, Foley upgraded the method to a minimally invasive TLIF, which has become popular as a minimally invasive lumbar fusion technique [54,66].

Artificial disc replacement

Compared to spinal fusion, artificial disc replacement allows for the maintenance of spinal mobility and range of motion. In the past two decades, there have been many artificial intervertebral discs designed, each intended for a specific spinal segment. The first lumbar disc replacement using a steel ball was created in 1966 by Fernström [67]. The ball was able to maintain disc height and motion, though long-term outcomes were poor [68]. In 1978, Fassio designed an elastic disc replacement that showed great improvement in shock absorption compared to Fernström’s ball.

Disc arthroplasty, or replacement, was not commercially available until the 1980s, with the creation of articulating nonelastic devices [68]. The SB Charité, a polyethylene-on-metal design, was one of the first lumbar implants available in 1982. Limitations in design and functionality have been addressed through model updates to Charité II and III [69]. Artificial discs are generally built with an endplate composed of cobalt-chrome, stainless steel, titanium, ceramic, or metal composite [69. Many devices have been marketed that involve metal-on-metal, artificial joint capsules, and elastic disc insertions. Recent developments revolve around creating prosthetic nucleus pulposus replacements. Ray, in 2002, published his prosthetic disc-nucleus device composed of hydrogel pellets in a polyethylene jacket [70]. Tissue engineering and the use of alternative biomaterials to create a functional replacement for intervertebral discs are currently underway [71].

Integration of robotics and navigation

To date, there are three robotic systems approved by the FDA for spine surgery. The SpineAssist (Mazor Robotics, Caesarea, Israel) became the first FDA-approved robot for spine surgery in 2004 [72]. It was followed by the ExcelsiusGPS (Globus Medical, Audubon, PA) and ROSA Spine (Zimmer Biomet, Warsaw, IN) more than 10 years later [72]. The SpineAssist has been the most studied, having been used for much longer. It operates as a bone-mounted robotic system with six degrees of freedom. Pre-operative computed tomography (CT) is necessary, allowing for robot-guided trajectory for accurate drilling during operation.

SpineAssist has been extensively studied in assisting with pedicle screw insertion; however, it has also been used increasingly in the assistance of vertebral body augmentation needle placement and biopsy or excision of osteoid osteoma [73]. A meta-analysis measuring the accuracy of pedicle screw placement by SpineAssist measured a significant increase in accuracy compared to manual screw placement [73]. D'Souza et al. have since updated SpineAssist with the release of Renaissance in 2011 and MazorX in 2016 [74].

**Figure 2:**
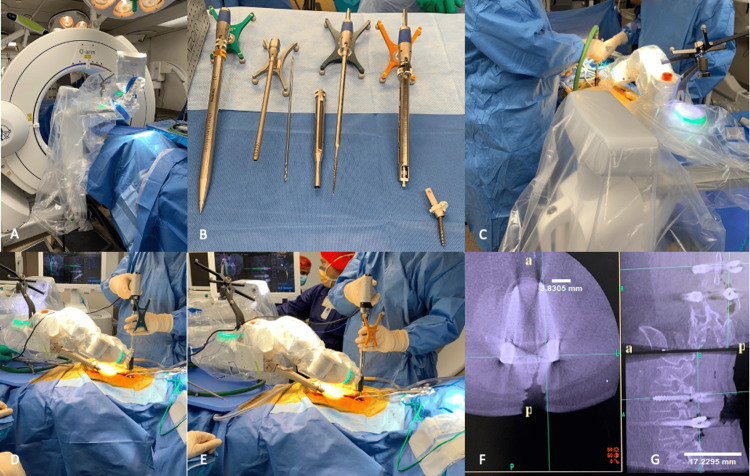
Medtronic Mazor X Stealth Edition intraoperative use during posterior lumbar pedicle screw placement. (A) Room staging with O-Arm Surgical Imaging System used for intraoperative 2D/3D imaging for integration into the Mazor system and placement of fluoroscopy on the opposing side of the patient for seamless access and transition intraoperatively. (B) From left to right: dilator, midas, drill bit, midas cannula, tap, solera, and MAS driver. The (C) drill, (D) tap, and (E) solera are used sequentially for screw placement. (F) Intraoperative fluoroscopy confirms accurate screw placement on axial imaging. (G) Intraoperative fluoroscopy confirms accurate screw placement on sagittal imaging. In figures F and G, "a" and "p" indicate anterior and posterior orientation, respectively. 'Image Credits: Tania Mamdouhi'.

The use of navigation has become exceedingly common in spine surgery. Most navigation systems are CT-based, such as the Aire Mobile (Brainlab), Stealth Station with O-Arm System (Medtronic, Minneapolis, MN), and Stryker Spinal Navigation System (Stryker, Kalamazoo, Michigan) (Figure 2). The major advantage of the utilization of robotics and navigation in spine surgery is increased accuracy in localization in real-time. Patients and staff are exposed to less radiation in SpineAssist procedures using the mini-open technique [75]. High initial costs and a lack of tactile feedback are major limitations.

Limitations and future directions

There are some limitations in this review that may have introduced potential bias. Some of the included studies were performed on small patient samples, which could have skewed results and called into question the general applicability of the surgical techniques and devices. While the authors believe this review to be all-encompassing, there may be other relevant examples of historical literature illustrating surgical spine applications that did not result in our preliminary search. 

Technological advancement in the field of spine surgery is a major area of focus. The evolution of surgical approaches to incorporate robotics is important for decreasing operating times and overall surgical complications. Smaller incisions can improve post-operative recovery and minimize infection risk.

Alternative biomaterials to meet the needs of evolving procedures are warranted. Materials must be biocompatible, resistant to microorganisms, and provide spinal stability while concurrently having longevity and low rates of fatigue. Copper was the major metal used historically for medical instrumentation and implantation due to its malleability and antimicrobial properties. The use of stainless steel in rods and screws was popular in the 1900s due to its strength and ability to be alloyed with other metals [76]. Corrosion and poor biocompatibility were major disadvantages to the use of both copper and stainless steel. Titanium, ceramic, cobalt chrome, nitinol, tantalum, and polyetheretherketone (PEEK) are the most common materials used today [76]. Titanium gained popularity due to its biocompatibility, similar biomechanical properties to cortical bone, and flexibility. Whereas PEEK has been shown to favor fibrous tissue formation, the inflammatory microenvironment of titanium alloy implants favors osteoblastic differentiation and, thus, bone formation [77]. Developments have been focused on novel coatings for existing materials that increase resistance to pull-out and optimize bony surface area interaction [76]. Coating pedicle screws with carbonated apatite cancellous bone cement has been shown to increase pull-out resistance by 68% and improve biomechanical performance [78].

Autologous bone grafting was the original implant of choice for stimulating bony fusion, though high rates of failure necessitated a more effective alternative. One of the most exciting developments in biomaterials is bone morphogenic protein (BMP), originally discovered in 1965 by Urist. BMP acts as an osteogenic agent, potentially eliminating the need for bone grafting. Recombinant human bone morphogenic protein-2 was given approval by the FDA only for anterior lumbar interbody fusion, where it is implanted within a titanium tapered cage [79]. Off-label use of BMP became widespread, with one study identifying an increase in use in fusion surgeries from 0.69% in 2002 to nearly 25% in 2006 in the United States [79]. Off-label use involves complicated tracking of surgical outcomes and complication rates. Reported rates of ectopic bone formation and overall increases in hospital charges have amplified apprehensions, in addition to overall ethical concerns [79]. Clinical studies have shown evidence of superior fusion success with BMP as compared to autografts, yet there are varied rates of complications in different surgical contexts [80-82]. Further exploration into the use of BMP in spine surgery will clarify its role in future practices.

## Conclusions

This review encapsulates the long history of spine surgery, subdivided by surgical approaches, implant inventions, and the more modern involvement of robotics and navigation. Throughout the manuscript, we provide uniquely succinct and thorough explanations of the development of several vital aspects of the specialty that have furthered its progress in the modern surgical climate. Knowledge of the history of spinal medicine and how it surfaced as both a neurosurgical and orthopedic subspecialty is important for understanding the origins of spinal therapies used today. Just as traction and bracing techniques revolutionized management for spinal deformities in Hippocrates’ era, minimally invasive surgery, motion preservation, and robotic modalities have commenced the new frontier for surgical practices of the future. We have provided a detailed course on the advancements and shortcomings of devices, techniques, and operative management for the treatment of all levels of the spine, making this review distinctly comprehensive. This expansive historical review highlights history’s role in the discovery of today’s techniques and tomorrow’s innovations.

## References

[1] Goodrich JT (2004). History of spine surgery in the ancient and medieval worlds. Neurosurg Focus.

[2] Marketos SG, Skiadas PK (1999). The modern hippocratic tradition. Some messages for contemporary medicine. Spine (Phila Pa 1976).

[3] Tarpada SP, Morris MT, Burton DA (2017). Spinal fusion surgery: a historical perspective. J Orthop.

[4] Vasiliadis ES, Grivas TB, Kaspiris A (2009). Historical overview of spinal deformities in ancient Greece. Scoliosis.

[5] Ramos G (2004). Efficacy of vertebral axial decompression on chronic low back pain: study of dosage regimen. Neurol Res.

[6] Fayssoux RS, Cho RH, Herman MJ (2010). A history of bracing for idiopathic scoliosis in North America. Clin Orthop Relat Res.

[7] Marketos SG, Skiadas PK (1999). Galen: a pioneer of spine research. Spine (Phila Pa 1976).

[8] Khan MJ, Srinivasan VM, Jea AH (2016). The History of bracing for scoliosis. Clin Pediatr (Phila).

[9] Kohler R (2010). Nicolas Andry de Bois-Regard (Lyon 1658-Paris 1742): the inventor of the word "orthopaedics" and the father of parasitology. J Child Orthop.

[10] Zampini JM, Sherk HH, Lewis A (2008). Sayre: the first Professor of Orthopaedic Surgery in America. Clin Orthop Relat Res.

[11] Golding-Bird CH (1879). Short notes on the use of Sayre's swing and plaster jacket in spinal disease. Br Med J.

[12] Hayward G (1815). An account of a case of fracture and dislocation of the spine. N Engl J Med.

[13] Markham JW (1952). The history of laminectomy prior to 1866. B Hist Med.

[14] Elsberg CA (1926). Tumors of the spinal cord and the symptoms of irritation and compression of the spinal cord and nerve roots; pathology, symptomatology, diagnosis, and treatment. Ann Surg.

[15] Hirabayashi K, Watanabe K, Wakano K, Suzuki N, Satomi K, Ishii Y (1983). Expansive open-door laminoplasty for cervical spinal stenotic myelopathy. Spine (Phila Pa 1976).

[16] Denaro V, Di Martino A (2011). Cervical spine surgery: an historical perspective. Clin Orthop Relat Res.

[17] Hirabayashi K, Satomi K (1988). Operative procedure and results of expansive open-door laminoplasty. Spine (Phila Pa 1976).

[18] Bacigaluppi S, Bragazzi NL, Martini M (2020). Fedor Krause (1857-1937): the father of neurosurgery. Neurosurg Rev.

[19] Truumees E (2015). A history of lumbar disc herniation from Hippocrates to the 1990s. Clin Orthop Relat Res.

[20] Mixter WJ, Barr JS (1934). Rupture of the intervertebral disc with involvement of the spinal canal. N Engl J Med.

[21] Walker CT, Kakarla UK, Chang SW, Sonntag VK (2019). History and advances in spinal neurosurgery. J Neurosurg Spine.

[22] Rogers WA (1942). Treatment of fracture-dislocation of the cervical spine. J Bone Jt Surg.

[23] Callahan RA, Johnson R, Margolis R, Keggi K, Albright J, Southwick W (1977). Cervical facet fusion for control of instability following laminectomy. J. Bone Joint Surg. Am.

[24] Hibbs RA (1912). A further consideration of an operation for Pott's disease of the spine: with report of cases from the service of the New York Orthopedic Hospital. Ann Surg.

[25] Hibbs RA, Risser JC, Ferguson AB (1931). Scoliosis treated by the fusion operation an end-result study of three hundred and sixty cases. J Bone Jt Surg.

[26] Gallie WE (1939). Fractures and dislocations of the cervical spine. Am J Surg.

[27] Omeis I, DeMattia JA, Hillard VH, Murali R, Das K (2004). History of instrumentation for stabilization of the subaxial cervical spine. Neurosurg Focus.

[28] Smith GW, Robinson RA (1958). The treatment of certain cervical-spine disorders by anterior removal of the intervertebral disc and interbody fusion. J Bone Joint Surg Am.

[29] Cloward RB (2007). The anterior approach for removal of ruptured cervical disks. J Neurosurg Spine.

[30] King D (1948). Internal fixation for lumbosacral fusion. J Bone Jt Surg.

[31] Kabins MB, Weinstein JN (1991). The history of vertebral screw and pedicle screw fixation. Iowa Orthop J.

[32] Boucher HH (1959). A method of spinal fusion. J Bone Joint Surg Br.

[33] Moftakhar R, Trost GR (2004). Anterior cervical plates: a historical perspective. Neurosurg Focus.

[34] Desai SK, Brayton A, Chua VB, Luerssen TG, Jea A (2013). The lasting legacy of Paul Randall Harrington to pediatric spine surgery: historical vignette. J Neurosurg Spine.

[35] Harrington PR (1962). Treatment of scoliosis. Correction and internal fixation by spine instrumentation. J Bone Jt Surg.

[36] Harrington PR, Tullos HS (1969). Reduction of severe spondylolisthesis in children. South Med J.

[37] Houten JK, Errico TJ (2005). Chapter 2 - History of spinal instrumentation: the modern era. Benzel's Spine Surgery.

[38] Hopf CG, Eysel P, Dubousset J (1997). Operative treatment of scoliosis with Cotrel-Dubousset-Hopf instrumentation. New anterior spinal device. Spine (Phila Pa 1976).

[39] Roy-Camille R (1989). Internal fixation of the unstable cervical spine by a posterior osteosynthesis with plates and screws. The Cervical Spine.

[40] Magerl F, Grob D, Seemann P (1987). Stable dorsal fusion of the cervical spine (C2-Th1) using hook plates. Cervical Spine.

[41] Goel A, Laheri V (1994). Plate and screw fixation for atlanto-axial subluxation. Acta Neurochir (Wien).

[42] Goel A, Desai KI, Muzumdar DP (2002). Atlantoaxial fixation using Plate and screw method: a report of 160 treated patients. Neurosurgery.

[43] Harms J, Melcher RP (2001). Posterior C1-C2 fusion with polyaxial screw and rod fixation. Spine (Phila Pa 1976).

[44] Mummaneni PV, Haid RW, Rodts GE (2004). Lumbar interbody fusion: state-of-the-art technical advances. Invited submission from the Joint Section Meeting on Disorders of the Spine and Peripheral Nerves, March 2004. J Neurosurg Spine.

[45] Matur AV, Mejia-Munne JC, Plummer ZJ, Cheng JS, Prestigiacomo CJ (2020). The history of anterior and lateral approaches to the lumbar spine. World Neurosurg.

[46] Capener N (1932). Spondylolisthesis. http://10.1002/bjs.1800197505.

[47] Burns B (1933). An operation for spondylolisthesis. Lancet.

[48] Bassani R, Gregori F, Peretti G (2019). Evolution of the anterior approach in lumbar spine fusion. World Neurosurg.

[49] Ito H, Tsuchiya J, Asami G (1934). A new radical operation for Pott's disease: report of ten cases. J. Bone Jt. Surg.

[50] Mercer W (1936). Spondylolisthesis: with a description of a new method of operative treatment and notes of ten cases. Edinb Med J.

[51] Jaslow IA (1946). Intercorporal bone graft in spinal fusion after disc removal. Surg Gynecol Obstet.

[52] Capener N (1954). The evolution of lateral rhachotomy. J Bone Joint Surg Br.

[53] Obenchain TG (1991). Laparoscopic lumbar discectomy: case report. J Laparoendosc Surg.

[54] Momin AA, Steinmetz MP (2020). Evolution of minimally invasive lumbar spine surgery. World Neurosurg.

[55] Pimenta L (2001). Lateral endoscopic transpsoas retroperitoneal approach for lumbar spine surgery. Brazilian Spine Society.

[56] Ozgur BM, Aryan HE, Pimenta L, Taylor WR (2006). Extreme lateral interbody fusion (XLIF): A novel surgical technique for anterior lumbar interbody fusion. Spine J.

[57] Mobbs RJ, Phan K, Malham G, Seex K, Rao PJ (2015). Lumbar interbody fusion: techniques, indications and comparison of interbody fusion options including PLIF, TLIF, MI-TLIF, OLIF/ATP, LLIF and ALIF. J Spine Surg.

[58] Mayer HM (1997). A new microsurgical technique for minimally invasive anterior lumbar interbody fusion. Spine (Phila Pa 1976).

[59] Thongtrangan I, Le H, Park J, Kim DH (2004). Minimally invasive spinal surgery: a historical perspective. Neurosurg Focus.

[60] Suezawa Y, Schreiber A (1988). [Percutaneous nucleotomy with discoscopy. 7 years' experience and results]. Z Orthop Ihre Grenzgeb.

[61] Yasargil MG (1977). Microsurgical operation of herniated lumbar disc. Advances in Neurosurgery.

[62] Caspar W (1977). A new surgical procedure for lumbar disc herniation causing less tissue damage through a microsurgical approach. Advances in Neurosurgery.

[63] Foley KT, Smith M (1997). Microendoscopic discectomy. Tech Neurosurg Neurol.

[64] Harms JG, Jeszenszky D (1998). The unilateral, transforaminal approach for posterior lumbar interbody fusion. J Orthop Trauma.

[65] Wiltfong RE, Bono CM, Charles Malveaux WM, Sharan AD (2012). Lumbar interbody fusion: review of history, complications, and outcome comparisons among methods. Curr Orthop Pract.

[66] Foley KT, Holly LT, Schwender JD (1976). Minimally invasive lumbar fusion. Spine.

[67] Fernström U (1966). Arthroplasty with intercorporal endoprothesis in herniated disc and in painful disc. Acta Chir Scand Suppl.

[68] Bono CM, Garfin SR (2004). History and evolution of disc replacement. Spine J.

[69] Othman YA, Verma R, Qureshi SA (2019). Artificial disc replacement in spine surgery. Ann Transl Med.

[70] Ray CD (2002). The PDN prosthetic disc-nucleus device. Eur Spine J.

[71] Iatridis JC, Nicoll SB, Michalek AJ, Walter BA, Gupta MS (2013). Role of biomechanics in intervertebral disc degeneration and regenerative therapies: what needs repairing in the disc and what are promising biomaterials for its repair?. Spine J.

[72] Vo CD, Jiang B, Azad TD, Crawford NR, Bydon A, Theodore N (2020). Robotic spine surgery: current state in minimally invasive surgery. Global Spine J.

[73] Devito DP, Kaplan L, Dietl R (2010). Clinical acceptance and accuracy assessment of spinal implants guided with SpineAssist surgical robot: retrospective study. Spine (Phila Pa 1976).

[74] D'Souza M, Gendreau J, Feng A, Kim LH, Ho AL, Veeravagu A (2019). Robotic-assisted spine surgery: history, efficacy, cost, and future trends. Robot Surg.

[75] Lieberman IH, Hardenbrook MA, Wang JC, Guyer RD (2012). Assessment of pedicle screw placement accuracy, procedure time, and radiation exposure using a miniature robotic guidance system. J Spinal Disord Tech.

[76] Warburton A, Girdler SJ, Mikhail CM, Ahn A, Cho SK (2020). Biomaterials in spinal implants: a review. Neurospine.

[77] Olivares-Navarrete R, Hyzy SL, Slosar PJ, Schneider JM, Schwartz Z, Boyan BD (2015). Implant materials generate different peri-implant inflammatory factors: poly-ether-ether-ketone promotes fibrosis and microtextured titanium promotes osteogenic factors. Spine (Phila Pa 1976).

[78] Lotz JC, Hu SS, Chiu DF, Yu M, Colliou O, Poser RD (1997). Carbonated apatite cement augmentation of pedicle screw fixation in the lumbar spine. Spine (Phila Pa 1976).

[79] Cahill KS, Chi JH, Day A, Claus EB (2009). Prevalence, complications, and hospital charges associated with use of bone-morphogenetic proteins in spinal fusion procedures. JAMA.

[80] Even J, Eskander M, Kang J (2012). Bone morphogenetic protein in spine surgery: current and future uses. J Am Acad Orthop Surg.

[81] Burkus JK, Sandhu HS, Gornet MF (2006). Influence of rhBMP-2 on the healing patterns associated with allograft interbody constructs in comparison with autograft. Spine (Phila Pa 1976).

[82] Hustedt JW, Blizzard DJ (2014). The controversy surrounding bone morphogenetic proteins in the spine: a review of current research. Yale J Biol Med.

